# Identification of a Hyperparasitic *Simplicillium obclavatum* Strain Affecting the Infection Dynamics of *Puccinia striiformis* f. sp. *tritici* on Wheat

**DOI:** 10.3389/fmicb.2020.01277

**Published:** 2020-06-24

**Authors:** Ning Wang, Xin Fan, Shan Zhang, Bo Liu, Mengying He, Xianming Chen, Chunlei Tang, Zhensheng Kang, Xiaojie Wang

**Affiliations:** ^1^State Key Laboratory of Crop Stress Biology for Arid Areas and College of Plant Protection, Northwest A&F University, Shaanxi, China; ^2^State Key Laboratory for Biology of Plant Disease and Insect Pests, Institute of Plant Protection, Chinese Academy of Agricultural Sciences, Beijing, China; ^3^USDA-ARS, Wheat Genetics, Physiology, Quality, and Disease Research Unit and Department of Plant Pathology, Washington State University, Pullman, WA, United States

**Keywords:** wheat stripe rust, *Puccinia striiformis*, *Simplicillium obclavatum*, hyperparasite, biological control

## Abstract

Wheat stripe rust, caused by *Puccinia striiformis* f. sp. *tritici* (*Pst*), is one of the most serious threats to wheat production worldwide. Changes of *Pst* virulence may circumvent resistance in wheat varieties, and application of fungicides may cause environmental problems. Parasites of *Pst* can be used to develop biological agents for environmentally friendly control of this fungal disease. Here, we report a hyperparasitic fungus isolated from *Pst* and identified it as *Simplicillium obclavatum* through molecular and morphological characterizations. We demonstrated that inoculation of *Pst*-infected wheat leaves with *S*. *obclavatum* reduced the production and germination rate of *Pst* urediniospores. Therefore, *S. obclavatum* has the potential to be developed into a biological control agent for managing wheat stripe rust.

## Introduction

Cereal rust outbreaks have caused significantly economic losses throughout the history of grain production. Almost all important grain crops, including wheat, rye, barley, oat, and other cereal crops, can be infected by rust fungi (Saari and Prescott, 1985). Furthermore, global grain production and worldwide food security are still severely challenged by cereal rust diseases (Fisher et al., 2012). Thus, development of tactics for control of cereal rust diseases is essential for food security. Biological control is one of the most environmentally friendly approaches for controlling plant pathogens but has not been intensively explored for managing cereal rusts.

Many biological antagonists can be effective against rust fungi (Bettiol and Várzea, 1992; Shiomi et al., 2006; Haddad et al., 2009; Jackson et al., 2012; Zheng et al., 2017). Fungi in several genera are hyperparasitic on rust fungi (Littlefield, 1981; Zheng et al., 2017). In the genus of *Cladosporium*, *C. uredinicola*, *C*. *cladosporioides*, *C*. *pseudocladosporioides*, *C*. *aecidiicola*, and *C*. *sphaerospermum* have been reported to parasitize rust fungi of the order Pucciniales (Keener, 1954; Srivastava et al., 1985; Vandermeer et al., 2009; Torres et al., 2017; Sun et al., 2019). Fungal species *C. cladosporioides*, *Eudarluca caricis*, *Microdochium nivale*, *Typhula idahoensis*, *Lecanicillium lecanii*, and *Alternaria alternata* have been reported to infect urediniospores of *Puccinia* (Littlefield, 1981; Nischwitz et al., 2005; Zhan et al., 2014; Zheng et al., 2017). For instance, *Lecanicillium lecanii* can parasitize the coffee rust pathogen, *Hemileia vastatrix*, reducing rust severity (Vandermeer et al., 2009; Jackson et al., 2012; Martins et al., 2015). *Simplicillium lanosoniveum* has been reported either as an entomopathogen or as a mycoparasite recovered from scale insects or rust fungal spores on coffee plants (Zare and Gams, 2001). As a biological control agent for soybean rust pathogen *Phakopsora pachyrhizi*, *S. lanosoniveum* was found to reduce the development of new uredinia and the germination rate of urediniospores (Ward et al., 2012). Baiswar et al. (2014) found that *S. lanosoniveum* also parasitizes rust pustules on *Elaeagnus latifolia*. Thus, biocontrol strategies have the potential for control of rusts.

One of the most important and destructive diseases on wheat is stripe rust, also called yellow rust, caused by *Puccinia striiformis* f. sp. *tritici* (*Pst*) (Chen, 2005; Wan et al., 2007). Yellow rust is named after yellow uredinia formed on leaf surfaces and other above ground parts of wheat. The previous studies reported that *M. nivale*, *L. lecanii*, *T. idahoensis*, *C. cladosporioides*, and *A. alternata* could parasitize *Pst* uredinia and urediniospores (Littlefield, 1981; Zhan et al., 2014; Zheng et al., 2017). We found that *Pst* uredinia were surrounded by white fungal hyphae during *Pst* urediniospore production, and white hyphae almost engulfed all *Pst* uredinia. Eventually, uredinia completely disappeared. Previous studies showed that hyperparasites stopped *Pst* sporulation (Zhan et al., 2014; Zheng et al., 2017).

Identification of parasites infecting cereal pathogenic fungi is essential for developing biological control strategies for managing plant diseases. In the present study, we report the discovery of a fungal strain isolated from *Pst*. We demonstrated that the fungus was able to parasitize the obligate biotrophic rust fungus. Through molecular and morphological characterizations, we identified the hyperparasitic fungus as species *Simplicillium obclavatum*. Our experiments showed that *S. obclavatum* was able to impair *Pst* sporulation and also reduce urediniospore germination. Therefore, the isolate of *S. obclavatum* has the potential to be developed as a biological control agent for managing stripe rust.

## Materials and Methods

### Isolation of a Hyperparasite From *Pst*-Infected Wheat Leaves

The mycoparasite samples consisting of several wheat leaves with abnormal *Pst* uredinia covered by white fungal mycelia were collected during our routine experiment in a growth chamber. The wheat leaves were from susceptible cultivar Fielder inoculated with urediniospores of *Pst* kept at 16°C and 80–90% relative humidity in a growth chamber. When *Pst* was sporulating 14 days after inoculation, white mycelia started appearing on *Pst* uredinia. The sample leaves were collected when white mycelia engulfed 50% uredinia 22 days after *Pst* inoculation. The leaf samples were treated with 75% alcohol for 1 min for surface disinfection, and the samples were washed with sterile water for five times.

The leaf samples were cut into 1–2-cm-long pieces, and the pieces were placed onto the potato dextrose agar (PDA) in Petri dishes that were incubated in darkness at 25°C for 5 days. Mycelial disks were cut from the edges of the fungal colony, transferred to new PDA dishes in a sterile inoculation chamber, and the new dishes were incubated under the same condition as described above for obtaining pure cultures. The purified isolates were kept on PDA slants at 4–8°C.

### Observation Using a Light Microscope

A mycelial plug (5 mm in diameter) from the edge of a growing colony was placed on to the center of a PDA plate and kept at 25°C for 10 days. The morphological characteristics of hyphae and conidia were observed using a light microscope (Olympus BX51T-32P01, Tokyo, Japan).

### Observation Using a Scanning Electron Microscopy (SEM)

Leaf samples bearing abnormal uredinia of *Pst* covered by fungal mycelia were harvested and surface-washed with distilled water. The samples were cut into 5 mm × 5 mm pieces and quickly placed in 4% glutaraldehyde fixative and fixed overnight at 4°C. The leaf samples were rinsed 4 times in 0.1 M pH 6.8 PBS buffer for 10 min each time and dehydrated with an ethanol series (30, 50, 70, 80, and 90%) for at least 15–30 min each at room temperature. The last 100% alcohol was taken three times for 30 min each time. The dehydrated samples were soaked in isoamyl acetate for 10–20 min and then processed with carbon dioxide drier, treated with spray-gold as previously described (Bomblies et al., 2008), and observed under a SEM (S-4800, Hitachi, Japan).

### Molecular Characterization

The parasitic isolate was inoculated onto the center of a PDA plate covered with a layer of cellophane and cultured in the dark at 25°C. Mycelia were collected five days post culture. After drying, the genomic DNA was extracted using the cetyltrimethylammonium bromide (CTAB) method (Rogers and Bendich, 1994). The internal transcribed spacer (*ITS1*) was used for identifying the hyperparasite. The polymerase chain reaction (PCR) solution in 20 μL volume comprised 1 μL 10 mM ITS primer for each (ITS1-Primer: TCCGTAGGTGAACCTGCG; ITS4-Primer: TCCTCCGCTTATTGATATGC) (White et al., 1990), 2 μl 0.4 mM dNTP, 10 μl PrimeSTAR^®^ Max DNA Polymerase (Takara, China, Dalian), 2 μL DNA solution, and 4 μL double-distilled water. Three other representative genes, elongation factor-1 alpha (*TEF1-*α), small subunit (SSU), and large subunit (LSU) of the ribosomal RNA gene, of *S*. *obclavatum* were used to confirm the ITS result. The hyperparasite DNA was amplified using specific primers of the *TEF1-*α (F: GCY CCYGGHCAYCGTGAYTTYAT; R: ATGACACCRACRGCRAC RGTYTG) (Rehner and Buckley, 2005), *SSU* (NS1: GTAGT CATATGCTTGTCTC; NS4: CTTCCGTCAATTCCTTTAAG (White et al., 1990), and *LSU* (LR0R: TACCTGGTTGATTCTGC; LR5: ATCCTGAGGGAAACTTC) (Vilgalys and Hester, 1990; Wei et al., 2019). All PCR reactions were performed as follows: denaturing at 95°C for 4 min, 40 cycles (from 95°C for 30 s, 60°C for 30 s, to extension at 72°C for 40 s), extension for 10 min at 72°C, and keeping at 16°C. PCR products were separated by 1.5% agarose gel electrophoresis. The PCR products were purified using the Gel Extraction Kit (CWBio, China, Beijing) and sequenced by Sangon Biotech (Shanghai, China).

### Phylogenetic Analysis

The sequences of *Lecanicillium* spp. and *Simpliciium* spp. were downloaded from the NCBI website^1^ (Table 1) and aligned by ClustalW in software MEGA7 using the default parameters (Kumar et al., 2016). The fungal isolates were clustered based on their ITS sequences using the neighbor-joining (NJ) method using MEGA7, and the branch robustness was determined using 1000 bootstrap replications (Kumar et al., 2016).

**TABLE 1:** Accession numbers of *Lecanicillium* spp. and *Simplicillium* spp. used in molecular characterization.

Species	Strain number	GenBank number	Substrate (including host)	Origin/locality
*L. wallacei*	CBS 101237	EF641891/IMI 331549	*Lepidopteran larva* on palm leaf	Indonesia
*L. antillanum*	CBS 350.85	NR_111097.1	Agaric	Cuba
*L. aphanocladii*	CBS 101286	AJ292430.1	*Agaricus bisporus*	United Kingdom
*L. lecanii*	CBS 101247	AJ292382/IMI 304807	Coccidae on Coffea	West Indies
*L. lecanii*	IMI 304817	AJ292383.1	*Cordyceps confragosa*	^–a^
*L. dimorphum*	CBS 363.86	NR_111101.1	*Heterodera* glycines cyst	Minnesota
*L. muscarium*	CBS 143.62	AJ292388/IMI068689	*Trialeurodes vaporariorum*	England
*L. muscarium*	IRAN 684C	EF641892.1/EF641854	*Akanthomyces muscarius*/*Pleurotus ostreatus*	Iran
*L. fungicola var. aleophilum*	CBS 357.80	AF324876.1	*Agaricus bitorquis*	Netherlands
*L. fungicola var. aleophilum*	CBS 300.70A	EF641885	Soil from rain forest	Queensland
*L. fungicola var. aleophilum*	CBS 171.80	EF641886.1	*Agaricus bitorquis*	Netherlands
*L. flavidum*	CBS 112974	EF641875	*Gomphidius glutinosus*	Finland
*L. flavidum*	CBS 300.70D	EF641877	*Coltricia perennis*	Austria
*L. flavidum*	CBS 290.80	EF641876	Decaying *Russula nigricans*	Germany
*L. fungicola var. fungicola*	CBS 992.69	NR_119653.1	*Agaricus bisporus*	Netherlands
*L. fungicola var. fungicola*	CBS 238.80	EF641880.1	Forest litter	United States
*L. fungicola var. fungicola*	CBS 696.88	EF641882	*Hypholoma capnoides*	Netherlands
*S. lamellicola*	CBS 116.25	AJ292393/AF339601	^–a^	-
*S. obclavatum*	CBS 311.74	EF468798/AJ292394/AF339567	Air, above sugarcane field	India
*S. obclavatum*	CBS 250.76	AY245654	Soil	India
*S. obclavatum*	CBS 510.82		Rust pustules on *Arachis hypogea*	Tamil Nadu
*S. lanosoniveum*	CBS 962.72	EF641862	-	-
*S. lanosoniveum*	CBS 704.86	AJ292396/AF339602	-	-
*S. lanosoniveum*	CBS 962.72	EF641862	-	-

*^*a*^Not available.*

### Hyperparasitism Assay

The inoculations were done following the previously described methods (Zheng et al., 2017). Two-week-old seedlings of wheat (cv. Fielder) were inoculated with urediniospores of *Pst* isolate V26 and incubated in a dew chamber at 12°C in the darkness for 24 h and then grown in a growth chamber at 16°C with a 16-h light photoperiod. Twelve days after *Pst* inoculation, plants were inoculated with the spore suspension (concentration 1.0 × 10^6^ spores⋅mL^–1^) of the identified *S. obclavatum* isolate and kept in a dew chamber at 16°C in the darkness for 24 h and then returned to the growth chamber for growing under the same conditions as described above. Plants inoculated with only *Pst* urediniospores were used as the control. Twelve days after *Pst* inoculation, symptoms were observed and yellow-colored uredinia were counted using pictures analyzed with software ImageJ number counting^2^. Samples were collected at 12, 36, 72, 96, 120, and 168 h after hyperparasite inoculation (hai) and observed with a SEM as described above. Urediniospores were collected at 3 and 5 days after hyperparasite inoculation (dai) for evaluation of the germination rate. The experiment included three biological repeats.

### *Pst* Biomass Analysis

Quantitative PCR (qPCR) was performed in a CFX96 Connect Real-Time PCR Detection System (Bio-Rad, Hercules, United States) to determine the *Pst* DNA content in the infected wheat leaves using a TB Green Premix DimerEraser (Perfect Real Time) (TaKaRa, Dalian, China). The fungal *Pst-EF1* (rust elongation factor 1) and wheat *TaEF1-*α (wheat elongation factor 1 alpha) fusion plasmids were diluted into a serial concentrations (10^3^, 10^4^, 10^5^, 10^6^, 10^7^, 10^8^, and 10^9^ fmol*cot*L^–1^) for generation of the standard curves. *Pst-EF1* primers (F: TGGTGTCATCAAGCCTGGTATGGT; R: ACTCAT GGTGCATCTCAACGGACT) and *Pst-EF1* primers (F: TTCG CCGTCCGTGATATGAGACAA; R: ATGCGTATCATGGTGGT GGAGTGA) were used for qPCR analysis. Genomic DNA of the infected wheat leaves was extracted from the infected leaves at 3, 5, 7, and 10 dai using the CTAB method as described above. Ct means were determined using qPCR. The *Pst* and wheat DNA concentrations were calculated according to the corresponding standard curves. The ratio of the *Pst* and wheat DNA concentrations was used to determine the *Pst* biomass. The experiment was conducted three times.

## Results

### Parasitization of *Pst* Uredinia by *S. obclavatum*

**FIGURE 1:**
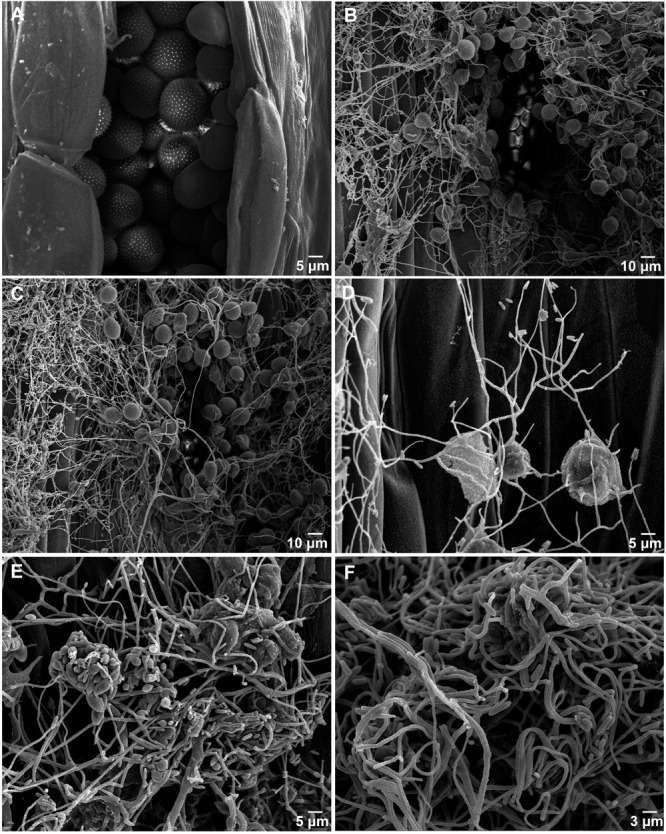
*Puccinia striiformis* f. sp. *tritici (Pst)* uredinia and urediniospores are parasitized by *Simplicillium obclavatum.* **(A)** Uredinia before being parasitized (×900). **(B)** Uredinia were shriveled at the early infection stage of the hyperparasitic process (×300). **(C,D)** The hyperparasite hyphae invading the urediniospores at the middle infection stage (×800). **(E,F)** The spores of *Pst* are completely swallowed at the late hyperparasitic stage (×1,500).

The morphological changes of *Pst* parasitized by the *S. obclavatum* isolate on wheat leaves were observed using a SEM. Urediniospores from normal uredinia were round or oval in shape (Figure 1A). When uredinia were parasitized by the hyperparasite, the urediniospores were shriveled at the early hyperparasitic infection stage (Figures 1B–D). The hyphae of the hyperparasitic fungus invaded *Pst* urediniospores at the middle infection stage (Figures 1C,D), and the urediniospores became completely swallowed at the late infection stage (Figures 1E,F). The destruction of *Pst* uredinial cell structures indicated that *Pst* uredinia parasitized by the hyperparasite lost viability.

### Identification of the Hyperparasite as *Simplicillium obclavatum*

**FIGURE 2:**
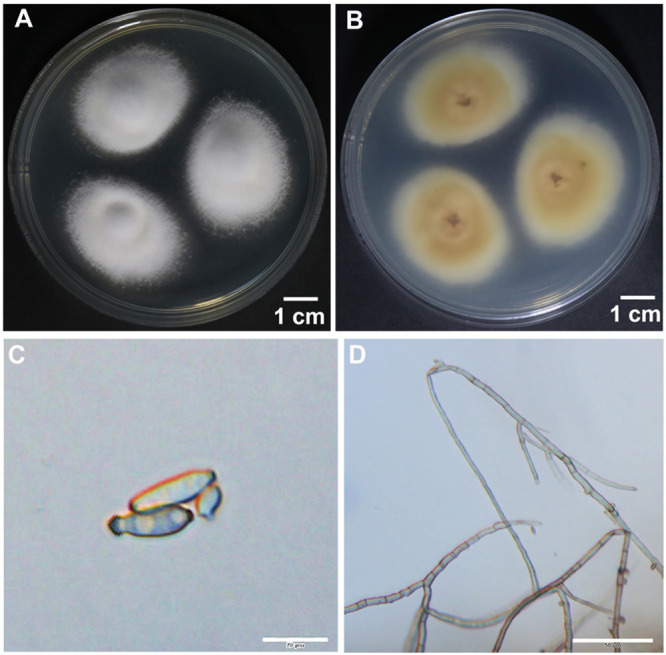
Morphological characteristics of hyperparasite *Simplicillium obclavatum*. **(A,B)** *S. obclavatum* cultured on PDA medium at 25°C for 10 days. **(A)** the front side; **(B)** the reverse side. **(C,D)** Conidia and septate hypha in morphology [bar = 20 μm in **(C)** and bar = 50 μm in **(D)**].

To identify the hyperparasite, we obtained an isolate named RV26 from hyperparasited *Pst* uredinia. The *in vitro* cultured RV26 colonies in PDA plates were white, floss in shape, and the reverse side was citron-yellow 10 days after in culture at 25°C (Figures 2A,B). The colonies were dense and reached 20–30 mm in diameter 10 days after in culture at 25°C (Figure 2A). Under an optical microscope, hyphae were septated, and conidia were typically obclavate to ellipsoidal, dark taupe, 20 to 35 μm × 2 to 5 μm in size (Figures 2C,D). These characteristics indicated that isolate RV26 is a filamentous fungus.

**FIGURE 3:**
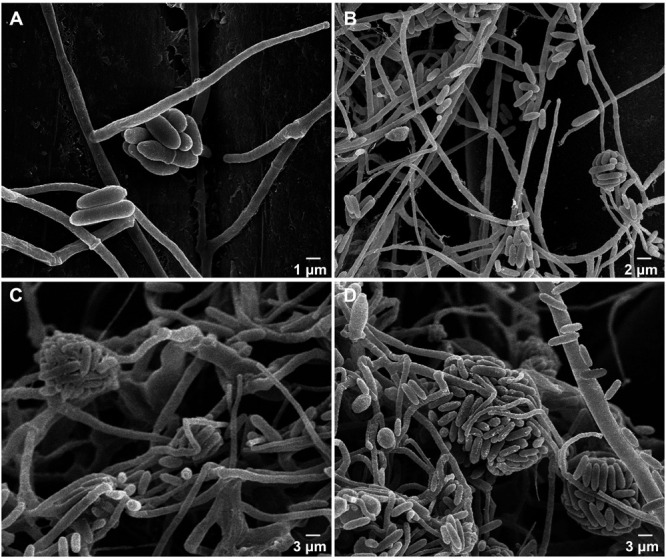
Morphologic characteristics of *Simplicillium obclavatum* under a scanning electron microscope. **(A–D)** Mycelium, conidia and conidium (×4,000, ×2,000, ×1,500, ×1,500, respectively).

The ultrastructure of RV26 was further examined using a SEM. Conidia were obclavate, had 3 or more spiral phialides, and were in imbricate chains with short imbricate chains on erected conidiophores (Figure 3). The morphological features of RV26 resembled *Simpliciium obclavatum* (Santana et al., 2017).

**FIGURE 4:**
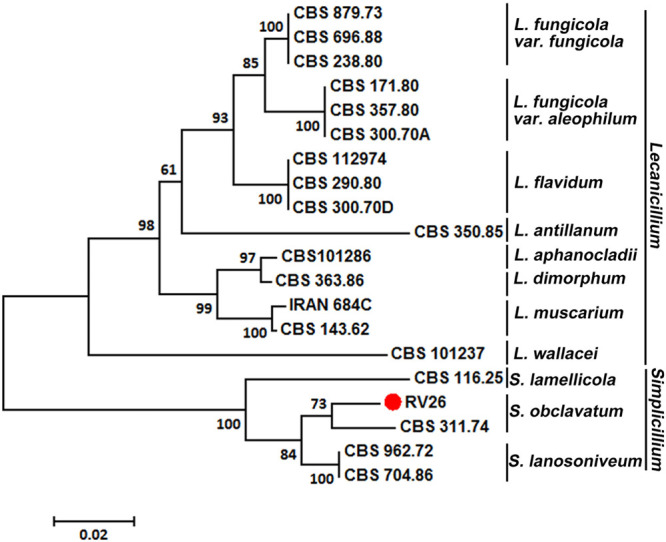
A phylogenetic tree constructed for species of *Verticillium*, *Lecanicillium*, and *Simpliciium* using software MEGA7 and the neighbor-joining (HJ) method. The hyperparasitic *S. obclavatum* isolate was marked with the red dot. The percentages of replicate trees in which the associated taxa clustered together in the bootstrap test (1000 replicates) are shown next to the branches.

The ITS1 region sequence of RV26 also supported the identification of the isolate as *S. obclavatum* (Figure 4). Additionally, the sequences of the RV26 fragments amplified with the *TEF1*α, *SSU*, and *LSU* primers of *S. obclavatum* confirmed RV26 as *S. obclavatum*. RV26 was clustered with other four *Simpliciium* species and was most close to *S. obclavatum* isolate CBS311.74 (Figure 4). Collectively, both the morphological and molecular characteristics supported the identification of the *Pst* hyperparasite as *S. obclavatum.*

### The Hyperparasitism of *S. obclavatum*

**FIGURE 5:**
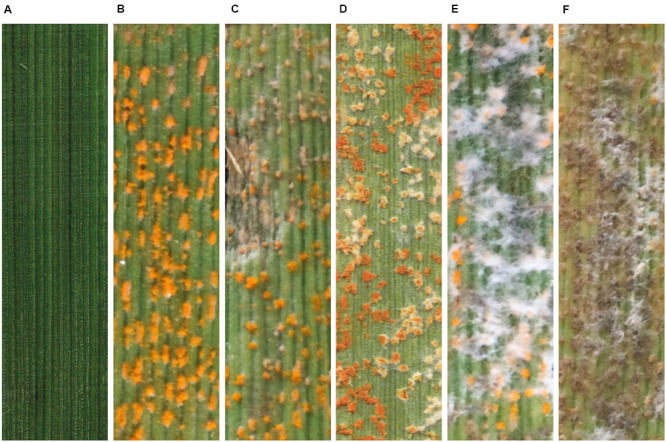
The pathogenicity test confirming the parasitism of *Simpliciium obclavatum* in *Puccinia striiformis* f. sp. *tritici* (*Pst*) grown on wheat leaves. **(A)** Wheat leaves inoculated with only the spore suspension of *Simpliciium obclavatum* 10 days post inoculation (dpi). **(B)** Wheat leaves inoculated with only *Pst* isolate V26 12 dpi. **(C–F)** Wheat leaves inoculated with the spore suspension of *S. obclavatum* 12 days after the inoculation with *Pst* V26, and **(C–F)** show symptoms and signs at 3, 5, 7, and 10 dpi with *S. obclavatum*, respectively.

To confirm the ability of *S. obclavatum* isolate RV26 to parasitize *Pst*, we performed a hyperparasitism assay. Wheat leaves inoculated with only the spore suspension of *S. obclavatum* showed normal growth at 22 dpi (Figure 5A), and those inoculated with only *Pst* race V26 had orange-colored normal *Pst* uredinia at 12 dpi (Figure 5B). In the test of the wheat leaves inoculated with the spore suspension of *S. obclavatum* isolate RV26 12 days after inoculation with *Pst* isolate V26, the hyperparasitic fungus started growing on *Pst* uredinia at 3 days after the inoculation with *S. obclavatum* and grew more rapidly 5, 7, and 10 days after the hyperparasite inoculation (Figures 5C,F).

**FIGURE 6:**
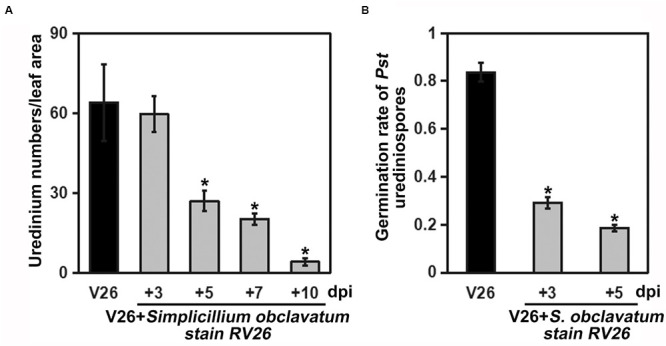
Quantification of *Puccinia striiformis* f. sp. *tritici* (*Pst*) uredinia and urediniospores viability. **(A)** The number of uredinia counted using an ImageJ number counter. The wheat leaves were first inoculated with *Pst* and 12 days after inoculated (dpi) with *Simpliciium obclavatum* conidia. Twelve days after *S. obclavatum* inoculation, the number of uredinium per cm^2^ of wheat leaf surface was calculated. **(B)** *Pst* urediniospore germination from leaves of non-treated and treated with *S. obclavatum. Pst* urediniospores of non-treated with *S. obclavatum* were used as the control.

**FIGURE 7:**
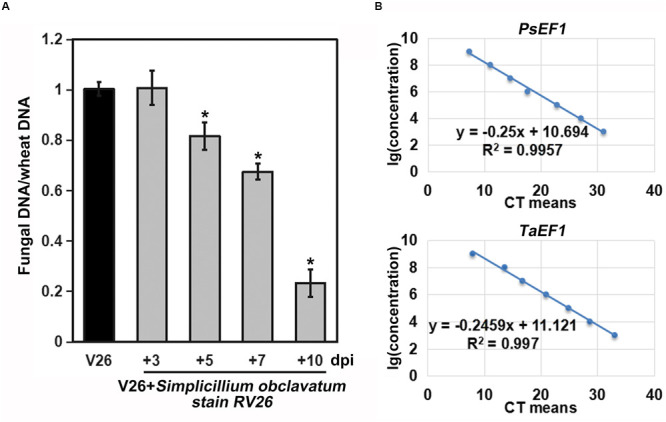
Biomasses of *Puccinia striiformis* f. sp. *tritici* (*Pst*) at different times after inoculation. **(A)** *Pst* biomass was calculated based on the ratio of *Pst* and wheat DNA concentrations. The relative quantities of PCR product of *TaEF1-*α and *PstEF1* in infected samples were calculated using the gene-specific standard curves to quantify *Pst* and wheat gDNA. **(B)** Standard curves derived from the serial dilutions. *PstEF1* (*Pst* elongation factor 1) and *TaEF1-*α (wheat elongation factor 1 alpha).

As *S. obclavatum* was growing and spreading, the number of *Pst* uredinia on the infected wheat leaves gradually decreased (Figure 6A). Moreover, *Pst* urediniospores collected from uredinia parasitized by RV26 showed a sharp loss of viability, indicated by the reduced rate of urediniospore germination (from 69% to 83%) (Figure 6B). It became unable to collect *Pst* urediniospores at 7 days after RV26 inoculation. The *Pst* biomass was decreased at more and more days after the inoculation (Figure 7A), which was also shown by the wheat and *Pst* standard curves (Figure 7B). These results indicated that *S. obclavatum* was able to parasitize *Pst* and effectively reduce the germination rate of *Pst* urediniospores.

**FIGURE 8:**
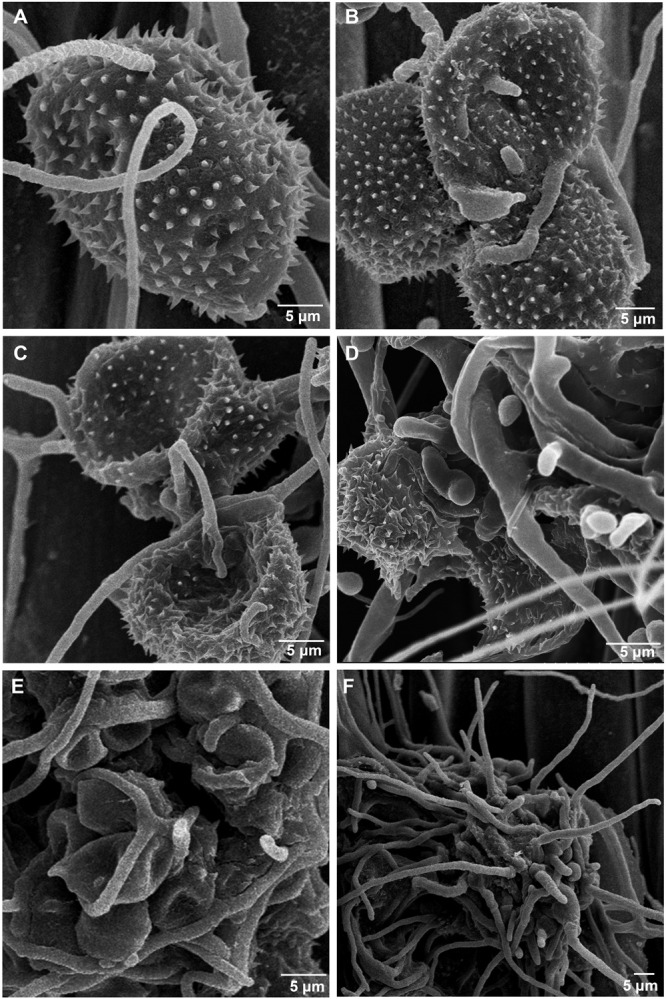
Morphologic characteristics of *Puccinia striiformis* f. sp. *tritici* (*Pst*) inoculated with *Simpliciium obclavatum* under a scanning electronic microscope. **(A)** Hyphae of *S. obclavatum* on the surface of a *Pst* urediniospore (×2,000) at 12 hai (hours after inoculation). **(B)** *S. obclavatum* generate germ tubes at 36 hai (×1,200). **(C,D)** *Pst* urediniospores surrounded by *S. obclavatum* hyphae. **(C)** at 72 hai (×1,800), and **(D)** at 96 hai (×3,000). **(E,F)**, *S. obclavatum* has colonized *Pst* urediniospores. **(E)** at 120 hai (×1,500) and **(F)** at 168 hai (×1,000).

To further confirm that the *S. obclavatum* isolate RV26 can effectively parasitize *Pst*, the morphologic characteristics of *Pst* inoculated with RV26 were observed using a SEM. *S. obclavatum* hyphae intruded into *Pst* urediospores through germ tubes (Figure 8A), gradually encroached *Pst* (Figures 8B,D), and destroyed *Pst* urediniospores at 5 d or 7 d after the parasite treatment (Figures 8E,F). These results confirmed that *S. obclavatum* RV26 were able to effectively parasitize *Pst*.

## Discussion

It is of great significance to study mycoparasites of cereal pathogenic fungi for the exploration of biological control of cereal diseases. Identification of mycoparasites is essential to understanding the biodiversity of hyperparasites. In the present study, we discovered that *S. obclavatum* isolate RV26 is able to parasitize obligate bitrophic pathogen *Pst.* This hyperparasite is able to reduce the development of *Pst* urediniospores, indicating that *S. obclavatum* has the potential to be used as a biocontrol agent to control *Pst*.

The colony morphology and conidial size are key factors in classifying *Lecanicillium* spp. and *Simplicillium* spp. (Zare and Gams, 2008; Wei et al., 2019). However, these morphological characteristics are considerably variable in different culture conditions and environments. Therefore, it is relatively difficult to identify fungi in these genera to the species level just based on the morphological characteristics. At first, we identified the isolate RV26 parasitizing *Pst* as *L. fungicola*, as it showed very similar morphological characteristics with *L. fungicola* (Zare and Gams, 2001). Although the genera *Simplicillium* spp. and *Lecanicillium* spp. are very closely related, conidial morphologies and ITS sequences are different (Zare and Gams, 2001, 2008). In the present study, we used both morphological and molecular characteristics to identify the RV26 isolate as *S. obclavatum*.

The effects of hyperparasites on plant pathogens are the basis for biological control of plant pathogenic fungi. Extensive studies have been conducted to identify and characterize hyperparasites of plant pathogens (Kohl et al., 2009; Berendsen et al., 2012). Previous studies have reported several hyperparasitic fungi parasitic on rust pathogens, such as *A*. *alternata*, *Aphanocladium album*, *Fusarium* spp., *Lecanicillium* spp., and *Scytalidium uredinicola* (Littlefield, 1981; Moricca et al., 2001; Koç and Défago, 2008; Tsuneda et al., 2011; Zheng et al., 2017). In the present study, we found that *S. obclavatum* could invade *Pst* as a hyperparasitic fungus.

Some hyperparasites have been used in managing plant diseases caused by fungi (Berendsen et al., 2012; Adhikari et al., 2014; Zhong et al., 2016). The *S. obclavatum* isolate RV26 identified in the present study is able to reduce or stop the growth of *Pst* urediniospores by growing into uredinia. This parasitic ability makes RV26 a potential biological control agent to remove *Pst* from infected wheat leaves. So far, it is clear that the *S. obclavatum* isolate is able to parasitize at the *Pst* sporulation stage, but it is unknown whether the fungus can infect in other *Pst* developmental stages. The parasitic fungus grows well under optimal humidity and temperature conditions for *Pst* to infect its wheat host. However, it is not clear what environmental factors affect the survival and parasitism ability of *S. obclavatum*. Therefore, further studies are needed to determine whether the parasitic fungus can be developed into a biological control agent for managing stripe rust under field conditions.

In summary, the present study identified *S. obclavatum* as a new hyperparasite of *Pst*. The fungus has the potential to be used as a biological control agent for control stripe rust. Additional research is needed to determine if the hyperparasite is environmentally friendly and, if so, to optimize conditions and develop techniques to produce and integrated the biological control agent into the current stripe rust management strategies and further to explore its potential to control other rust pathogens.

## Data Availability Statement

The raw data supporting the conclusions of this article will be made available by the authors, without undue reservation, to any qualified researcher.

## Author Contributions

XW, ZK, and NW conceived and designed the experiments. NW, XF, BL, and SZ conducted the experiments. NW, XF, and MH analyzed the data. NW, CT, and XW wrote the manuscript. XC helped revise the manuscript.

## Conflict of Interest

The authors declare that the research was conducted in the absence of any commercial or financial relationships that could be construed as a potential conflict of interest.

## Footnotes

1 https://www.ncbi.nlm.nih.gov/

2 https://imagej.nih.gov/ij/
